# The International Polar Year, 2007–2008, An Opportunity to Focus on Infectious Diseases in Arctic Regions

**DOI:** 10.3201/eid1401.071405

**Published:** 2008-01

**Authors:** Alan J. Parkinson

**Affiliations:** * Centers for Disease Control and Prevention, Anchorage, Alaska, USA

On 3 occasions over the past 125 years, scientists from around the world have worked together to organize scientific and exploration activities in polar regions (www.ipy.org). The first International Polar Year (IPY) in 1881–1884 marked the first major coordinated international scientific initiative to collect standardized meteorological and geophysical data in polar regions. Fifteen expeditions led by 12 nations amassed a large amount of data, but the scientific value was diminished by disjointed publication efforts and lack of long-term institutional commitment; lessons were learned and corrected in subsequent polar years. The second IPY began in 1932. Forty-four nations led expeditions in the Arctic and Antarctic, resulting in greater understanding of the aurora, magnetism, and meteorology. Air and marine navigation, radio operations, and weather forecasting were greatly improved as a result. The third IPY, in 1957–58, was renamed the International Geophysical Year and capitalized on technologic advances developed during World War II. Technologic and scientific momentum was redirected toward research, particularly to studies of the upper atmosphere, a legacy that continues to the present day. Notable achievements included launching the first satellite, measurement of atmospheric greenhouse gases, delineating the system of mid-ocean ridges, and confirming the theory of plate tectonics.

The current 4th IPY covers the period March 2007 to March 2009, although it is officially designated IPY 2007–2008. It was established by the International Council for Science, the National Academy of Sciences and World Meteorological Organization. This period of focused scientific research promises to “... further our understanding of the physical and social process in Polar Regions, examine their globally-connected role in the climate system and establish research infrastructure for the future, and serve to attract and develop a new generation of scientists and engineers with the versatility to tackle complex global issues” (www.ipy.org). The 2007–2008 IPY also features human health as a research theme for the first time and thus presents an opportunity to do the following: 1) increase global awareness and visibility of health concerns of Arctic peoples, 2) foster human health research, 3) promote health protection strategies, and 4) ultimately improve the health and well being of Arctic peoples (www.arctichealth.org/ahhi).

The Arctic is unique in many respects. It has a sparse population, scattered over a very large geographic area; climate and latitude marked by seasonal extremes of temperature and daylight; and a spirited history of cross-border cooperation on issues of concern to Arctic peoples. The Arctic is home to ≈4 million people; approximately one tenth (350,000) are of indigenous ancestry (*1*). Many live in remote, isolated communities and are, as depicted by Fred Machetanz on the cover of this issue, still dependent on a traditional subsistence way of life that has little economic infrastructure. Health concerns of Arctic peoples include the remaining health disparities that exist between indigenous and nonindigenous segments of the population as well as the potential impact of a changing Arctic environment, characterized by rapid economic change and modernization, environmental pollution, alterations in the traditional subsistence food supply, and climate change (*2*).

Life expectancy in Arctic populations has greatly improved since the last IPY. For example, in 1950, the life expectancy for Alaska Natives, the indigenous people of Alaska, was 47 years at birth compared with 66 years for the general US population. By 2000, the life expectancy for Alaska Natives was 69.5 years, a gain of >20 years. Reductions in deaths from infectious diseases for Alaska Natives have been especially dramatic. In 1950, 47% of deaths among Alaska Natives were due to infections, as compared with only 3% for non-Native Alaskans. By 1990, infectious diseases caused only 1.2% of Alaska Native deaths, very similar to the 1% seen for non-Native Alaskans. Much of this improvement can be attributed to improved living conditions, provision of safe water and sewage disposal, implementation of vaccination programs, training of community-based health providers, and an integrated healthcare delivery system that provides improved access to better quality healthcare (*3*).

Despite improvements in these health indicators of Arctic residents, life expectancy is shorter and infant mortality rates are higher among indigenous Arctic residents in the US Arctic, northern Canada, and Greenland when compared with those of nonindigenous residents of Arctic countries. For example, life expectancy of Alaska Natives still lags behind that of the general US population, which was 76.5 years in 2000. Similarly, indigenous residents of the US Arctic, northern Canada, and Greenland have higher mortality rates from injury and suicide and as well as higher hospitalization rates for infants with pneumonia, meningitis, and respiratory infections (*4**–**6*). Some infectious diseases are linked to cultural practices of the indigenous population, such as botulism from ingesting improperly prepared traditionally fermented foods (*7*) and trichinosis from consuming meats from land and marine mammals (L.N. Moller, unpub. data). Many of these infectious disease health disparities can be eliminated through the focused application of existing public health strategies.

Many communities that were once isolated are now linked to major cities by air transportation and are only an airplane ride away from more densely populated urban centers. Consequently, these communities are now vulnerable to the importation of new and emerging infectious diseases (such as influenza, severe acute respiratory syndrome [SARS] or SARS-like infectious diseases and antimicrobial drug–resistant pathogens such as multidrug-resistant *Streptococcus pneumoniae,* methicillin-resistant *Staphylococcus aureus*, and tuberculosis).

The changing climate is already affecting Arctic communities. It is increasingly apparent that the most vulnerable will be those living a traditional subsistence lifestyle in remote communities; they are already facing health or economic challenges. The melting permafrost, flooding, and storm surges are progressively destroying village sanitation and drinking water infrastructures of many Arctic communities, paving the way for outbreaks of food- and water-borne diseases and respiratory infections (*8*). In addition, climate change may drive increased dissemination of zoonotic pathogens in water- and food-borne pathways (*Giardia, Cryposporidium, Toxoplasma, Trichinella*, and *Echinococcus* species), posing a direct threat to human health in communities that rely on wildlife as a source of food.

Temperature and humidity markedly affect the distribution, density, and behavior of many arthropod vectors and may increase the incidence and expand the northern range of many vector-borne diseases such as West Nile virus (*8*). Specific stages of the life cycles of many helminths and arthropods may be greatly influenced by temperature (*9*). For example, small changes in temperature can substantially alter the transmission of lung worms and muscle worms pathogenic to ungulates (caribou, muskoxen, thinhorn sheep, and moose). In other parts of the world, the convergence of population dynamics, environmental factors, and animal reservoirs has resulted in dramatic outbreaks of apparently new infectious diseases that constitute a considerable threat to global human health (most recently, SARS and avian influenza). The full impact of climate change on these host-parasite interactions, animal health population dynamics, and human health is unknown, but the known effects of climate change on these systems underscores the need for close monitoring.

In recognition of IPY 2007–2008, this issue of Emerging Infectious Diseases highlights infectious disease challenges faced by residents of Arctic regions. The IPY is a unique opportunity to increase awareness and visibility of infectious disease concerns of Arctic peoples. It can serve to reinvigorate cross-border collaborative infectious disease research networks that will focus on eliminating remaining health disparities caused by infectious diseases in these populations (www.inchr.org). Finally, the IPY can increase focus on development of sustainable international surveillance networks across the Arctic for monitoring infectious diseases of concern and evaluating the effectiveness of current intervention strategies (*10*). The establishment of these networks will be essential for detecting the emergence of climate-sensitive infectious diseases in both human and wildlife populations and the design of effective interventions aimed at reducing risk and eliminating disease (*11*,*12*).
